# Flow Experiences During Visuomotor Skill Acquisition Reflect Deviation From a Power-Law Learning Curve, but Not Overall Level of Skill

**DOI:** 10.3389/fpsyg.2019.01126

**Published:** 2019-05-15

**Authors:** Benjamin Ultan Cowley, Jussi Palomäki, Tuisku Tammi, Roosa Frantsi, Ville-Pekka Inkilä, Noora Lehtonen, Pasi Pölönen, Juha Vepsäläinen, Otto Lappi

**Affiliations:** ^1^Cognitive Science, Department of Digital Humanities, Faculty of Arts, University of Helsinki, Helsinki, Finland; ^2^Helsinki Centre for Digital Humanities, Helsinki, Finland; ^3^Traffic Research Unit, TRUlab, University of Helsinki, Helsinki, Finland

**Keywords:** Flow, skill acquisition, power law of practice, visuomotor performance, steering, high performance cognition

## Abstract

Flow is a state of “optimal experience” that arises when skill and task demands match. Flow has been well studied in psychology using a range of self-report and experimental methods; with most research typically focusing on how Flow is elicited by a particular task. Here, we focus on how the experience of Flow changes during task skill development. We present a longitudinal experimental study of learning, wherein participants (*N* = 9) play a novel steering-game task designed to elicit Flow by matching skill and demand, and providing clear goals and feedback. Experimental design involves extensive in-depth measurement of behavior, physiology, and Flow self-reports over 2 weeks of 40 game trials in eight sessions. Here we report behavioral results, which are both strikingly similar and strong within each participant. We find that the game induces a near-constant state of elevated Flow. We further find that the variation in Flow across all trials is less affected by overall performance improvement than by deviation of performance from the expected value predicted by a power law model of learning.

## 1. Introduction

In many fields of human endeavor—such as music, art and sports—the skilful performance of a demanding task can elicit a state of “optimal experience” called Flow (Csikszentmihalyi, 1975). The Flow state is thought to be dependent on several pre-conditions for the eliciting task, and characterized by several phenomenological features (Nakamura and Csikszentmihalyi, 2002; Engeser and Schiepe-Tiska, 2012; Keller and Landhäußer, 2012). *Conditions* for Flow to occur define certain characteristics of the task and one's skill in the task: C1: challenge should match skill in a demanding task. C2: the task setting should present clear and personally significant goals. And C3 the setting should provide unambiguous feedback on goal achievement. When these conditions are met and the individual enters a mode of high performance, they may experience a set of phenomenological features characterizing “the Flow experience”: F1 total focus in the present moment, and concentration on what one is doing; F2 merging of action and awareness (“being one with the task”); F3 loss of reflective self-consciousness, a sense of effortlessness; F4 a sense of personal control and confidence in one's skill; F5 positive affect, the activity is experienced as highly enjoyable; F6 a distortion of temporal experience (time may seem to go slower or faster than normal). These *consistently co-occurring* features of the experience imply that Flow is *autotelic*, i.e., Flow-producing activities are intrinsically rewarding, people want to do them for their own sake regardless of external reward.

The antecedent conditions (C1-3) and phenomenological features (F1-6) of Flow have been investigated for several decades, mainly using analysis of self-report data from people engaged in natural everyday or expert performance (Csikszentmihalyi and Bennett, 1971; Moneta, 2012). Despite this, debate continues around the precise definition of the pre-conditions, especially C1 (challenge–skill balance).

At least three different models have described how Flow depends on challenge–skill ratio. Each model makes different assumptions for how this dependence is affected when skill and challenge change during learning. The original Flow model (Csikszentmihalyi, 1975) assumed that levels of challenge and skill can vary independently from low to high, and that Flow can happen when skill and challenge match at any level. This is the seminal Flow “channel” model.

The now-classic octant/quadrant models (Massimini et al., 1988) instead suggested that Flow only happens when skill and challenge exceed a certain threshold level. If the challenge or skill of task performance are too low, even if matched, then the task will not elicit Flow. At a certain point, the level of matched skills and challenges will become enough to elicit Flow; we will refer to this point as the *reference level*. The octant/quadrant models have been criticized because it is unclear how to determine the reference level. Does challenge need to exceed the challenge of most typical everyday tasks? Does the task need to be challenging for the individual, or relative to typical skills of a reference population? Or, does the reference level come from some universal benchmark of physical effort or information-processing complexity^1^? Challenge (and skill) could also be task-specific, with its reference level continually recalibrating to the reference level of past performance in the specific task. Each of these issues beg the question: what happens during task learning? Does the reference level need to be recalibrated to track the levels of skill and challenge as they increase?

In their critical examination of these models, Keller and Landhäußer (2012) argue that the concept of reference level is problematic, as it might not be empirically determinable for researchers, or even psychologically available to performing individuals (see also Moneta, 2012). Keller and Landhäußer (2012, p. 56) proposed the Flow intensity model, which has the dimensions “perceived challenge–skill balance” and “subjective value of the task” to define conditions that elicit Flow. This model has no reference levels. On the other hand, the intensity model takes no account of the *direction* of a challenge–skill imbalance, thus losing information which may be important to understand the Flow experience.

To sum up, the state of the art is unclear on three main points. First, it is unclear whether reference levels of challenge and/or skill govern the emergence of Flow. If they do, it is unclear how the levels are defined. If defined relative to a given task or task episode, we must ask: should the level change with skill acquisition? If defined relative to other tasks, we must ask: should the task demand be compared to the skill sets of the individual, or a reference population, or an absolute standard? Second, it is unclear if the direction of challenge–skill ratio is important, i.e., does it make a difference that skill exceeds challenge vs. challenge exceeds skill. Third and finally, these models capture a static snapshot of Flow. Thus, Flow research must still deal with the effects of *learning* on C1 (challenge–skill balance).

The matter is of importance because understanding how the Flow conditions behave across different levels of skill is relevant to any field interested in the development of performance (e.g., development of coaching practices or concentration techniques in sport Jackson and Marsh, 1996). Also, an understanding of the mechanisms that mediate Flow across a learning process could help to enhance enjoyment or performance through better design, e.g., of recreational tasks such as games (Chen, 2007). However, these aims call for studies of Flow elicited across different stages of learning, with a more controlled and quantitative approach. Such an approach can build on recent studies of Flow from fields of experimental psychology (Keller and Blomann, 2008; Harris et al., 2017) and psychophysiology (Peifer, 2012; Peifer et al., 2014; Harmat et al., 2015; Wolf et al., 2015; Labonté-LeMoyne et al., 2016).

Here we report an experimental skill-acquisition study on the connections between performance and the self-reported phenomenology of Flow. We introduce a novel, demanding visuomotor task. With a longitudinal design, we are afforded more power to examine the connections of Flow and performance, within- and between-subjects. We also model the learning shown by participants, finding good fit of the data to a power-law curve, which has been shown to closely approximate a very wide range of skill acquisition datasets (Newell and Rosenbloom, 1982; Logan, 1988; Palmeri, 1999).

Results show that our task successfully elicits an elevated level of Flow across all self-reports. Yet when we examine Flow with fine granularity, we see that variation in Flow responses relates less to overall performance improvement, than it does to deviation of performance from the expected value predicted by a power law model of learning, with higher performance associated with higher Flow and lower performance with lower Flow.

### 1.1. Protocol and Research Questions

**Figure 1:**
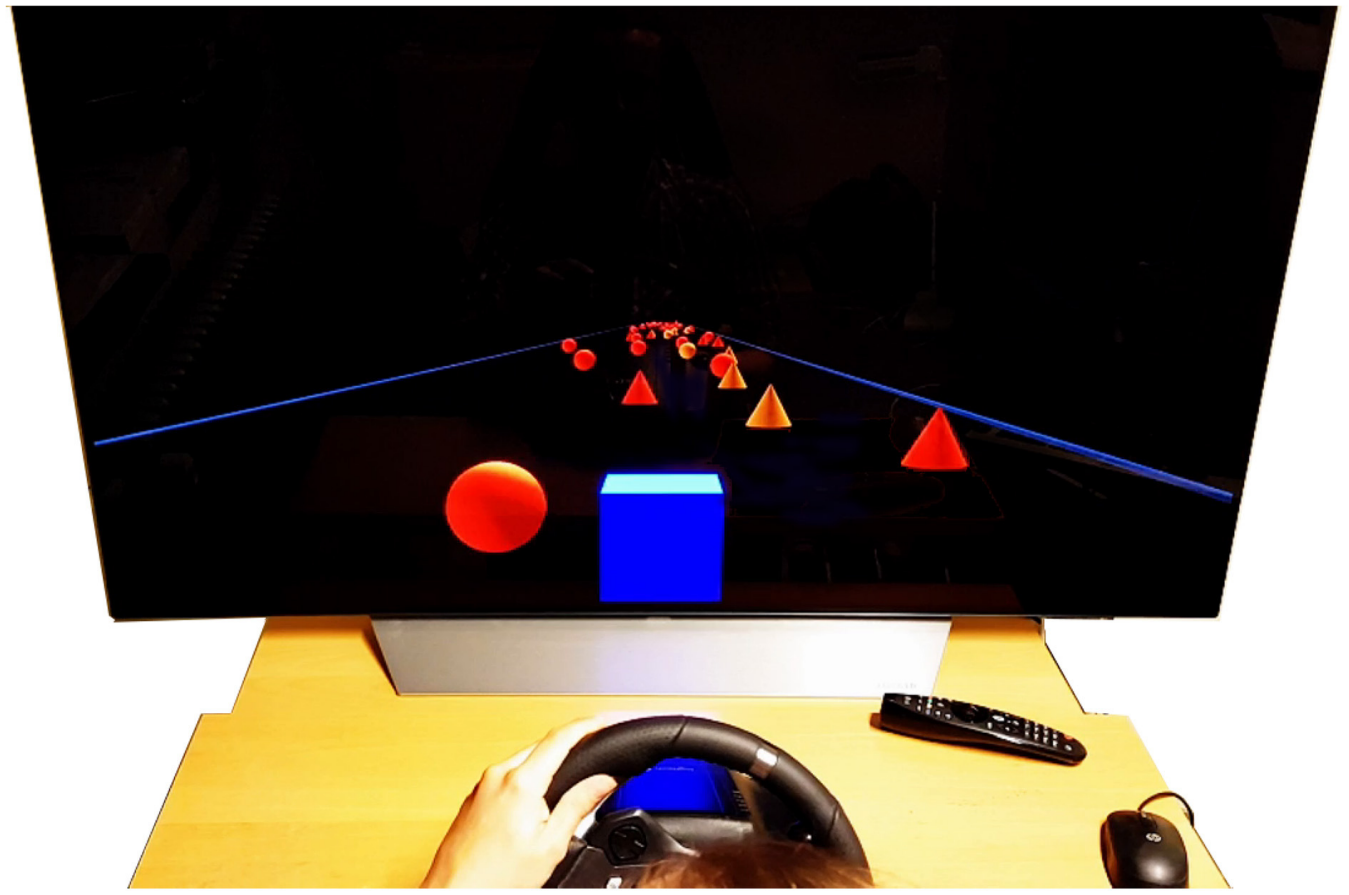
The high-speed steering task. The participant steers the blue cube to avoid conical/spherical obstacles on the track, which is bounded to each side by dark blue parallel lines. The game was designed to continually adapt the difficulty level (speed) to the participant's skill (obstacle collisions). Such balance is considered one of the key antecedents of Flow.

Participants learned to play a custom-made high-speed steering game (Figure 1^2^). The game was specifically designed to elicit Flow through balancing task demand with the skill level of the participant, and providing clear immediate feedback. The aim in the game was to steer a blue cube through a course with randomly placed red obstacles at the highest possible speed. The cube started each game at a fixed forward velocity, which increased at a constant rate. The lateral position of the cube was controlled by the steering wheel. Collision with obstacles reduced speed by a fixed amount, also indicated by a flashing of the screen (see section 2 for units).

This design, inspired by psychophysical staircase methods (Cornsweet, 1962), ensured constant match between skill and demand at the participant's level of performance. The performance was measured by duration of the trial (shorter duration = faster average speed = better), displayed as a time score at the end of each trial.

Participants played the game for forty trials across eight sessions, over a period of 2–3 weeks, which was sufficient to achieve good proficiency in this task with no ceiling effect. The 10 item Flow Short Scale (Engeser and Rheinberg, 2008) was filled after each trial to probe self-reported Flow in the task. Physiological data were recorded (skin conductance, heart rate, and eye tracking), during the task and a 5 min baseline, in sessions one and five-to-eight. This data introduces considerable further research questions and so falls outside the scope of this report.

This design allowed us to explore the following Research Questions:

1. RQ1. How does performance change over time, i.e., what is the shape of the “learning curve” (LC)? Specifically, does performance in the game improve, and does improvement follow a power law of practice (as found in much previous work on visuomotor skill acquisition Newell and Rosenbloom, 1982)?
2. RQ2. How is trial-wise self-reported Flow (from here on simply “Flow”) related to performance? Specifically, is performance improvement across the whole experiment (i.e., learning) accompanied by higher levels of Flow?

## 2. Methods

### 2.1. Participants

A convenience sample (*N* = 9, 6 males, 3 females) was recruited via student mailing lists at the University of Helsinki. The participants were between 22 and 38 years of age (mean 27, *SD* 3) with normal or corrected-to-normal visual acuity and no history of neurological or psychiatric disease.

Eight of the participants had a driving license; two participants reported <10,000 km lifetime kilometrage, three participants 10,000–30,000 km, two 30,000–100,000 km, and one participant >100,000 km. Two had no or very little previous gaming experience, two participants played 1–3 h a month, and five participants stated they play over 1 h a week. Table 1 shows the details of each participant.

**Table 1:** Participant background information.

**Participant**	**Gender**	**Age**	**Driving license**	**Driving experience (km)**	**Gaming experience**
1	M	≤27	Yes	1,000–10,000	At least 1 h a week
2	M	≤27	Yes	10,000–30,000	At least 1 h a week
3	F	≤27	No	0–1,000	None or very little
4	F	≥28	Yes	0–1,000	At least 1 h a week
5	M	≤27	Yes	30,000–100,000	At least 1 h a week
6	M	≤27	Yes	30,000–100,000	1–3 h a month
7	F	≥28	Yes	10,000–30,000	None or very little
8	M	≥28	Yes	100,000+	1–3 h a month
9	M	≤27	Yes	10,000–30,000	At least 1 h a week

All participants were naive about the specific hypotheses and purpose of the study, other than that the time of recruiting they were informed that the experiment was about game experience and learning. Participants were given 11 cultural vouchers (1 voucher is worth 5 euro) in compensation for their time. They were told that they would get 9 vouchers for participating in all sessions and 2 extra vouchers if they improved their performance in the game. The criteria for sufficient improvement were not stated explicitly, and in fact all participants were given the two extra vouchers.

Participants were briefed and provided written informed consent before entering the study, and were aware of their legal rights. The study followed guidelines of the Declaration of Helsinki and was approved by the University of Helsinki Ethical review board in humanities and social and behavioral sciences (statement 31/2017; study title MulSimCoLab).

### 2.2. Design

**Figure 2:**
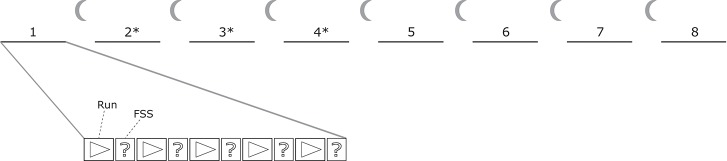
The game was played in eight sessions on eight different days. In sessions 1 and 5–8, physiological signals were recorded during task performance; in sessions 2–4(*) no physiology was recorded. Each session consisted of five trials (2–4 min) followed by a self-report questionnaire (FSS, Flow Short Scale) about the latest trial.

The experiment was divided into eight sessions, on eight different days over a period of 2–3 weeks scheduled at each participant's convenience. In each session, the participant played five trials of the driving game, each trial lasting 2–4 min depending on their performance, for approximately 15 min of driving time per session. The judgement of how much total playtime (here, 2 h) would be sufficient to develop good task proficiency was based on extensive informal piloting, including prior observations with other convenience samples. Figure 2 illustrates the protocol.

After each trial, the participant was shown the trial duration and the number of collisions, after which they filled in a self-report questionnaire (FSS). In sessions 1 and 5–8 (lasting approx. an hour), eye-tracking and physiological signals (electrodermal activity and heart rate) were recorded in a 5 min baseline recording before playing, and during gameplay. In sessions 2–4 (lasting 20–30 min), no physiological measurements were taken.

### 2.3. Materials

#### 2.3.1. Game

The experimental task was a custom-made high-speed steering game *CogCarSim* designed specifically for the study of Flow and coded in Python^3^.

The participant steered a cube “avatar” moving forward along a straight track bounded by edges that could not be crossed. The cube's side length was 2 units, and the track was 25 units wide. The horizontal field of view angle of the virtual camera was 60° and vertical 32°. The camera was positioned behind the cube at 4 units height, pointing forward along the track.

Stationary obstacles (red cones, red or yellow spheres with a height/diameter of 2 units) on the track had to be avoided. For each trial, a total of 2,000 obstacles were placed randomly on the track, with placement constrained to always allow a path through. Track length varied between 24196.4 and 24199.7 units (mean 24197.8, sd 0.8). The speed of the cube was initially set to 1.6 units per step (96 units per second); increased at a constant rate (0.0012 units/step at every step); and slowed down if obstacles were hit (0.102 units/step at each collision). When a collision caused a speed drop, the screen flashed to indicate a collision; there followed an immunity period of 100 steps during which additional collisions did not cause further speed drops. Participants could only affect speed indirectly, by avoiding collisions. Participants were instructed to avoid as many obstacles they could in order to complete the trial as fast as possible.

The game had maximally simple one degree-of-freedom linear and holonomic dynamics: the horizontal position of the cube was directly proportional to steering wheel angle. Extensive self-piloting was done to adjust the graphics, e.g., virtual eye height; plus starting and increment speeds, rate of change of speed during collisions, and steering wheel sensitivity (steering ratio and damping).

The participants started each trial by pressing a button on the steering wheel when they felt ready. At the end of each trial, the elapsed time and number of collisions were displayed, along with a high score of the participant's ten best trials so far.

Data collected by CogCarSim included the positions, shape, and color of obstacles on the track; trial-level aggregated performance data (trial duration, number of collisions, average velocity); and within-trial time series data (steering wheel and cube position, speed, registered collisions).

#### 2.3.2. Equipment

The game was run on a Corsair Anne Bonny with Intel i7 7700k processor and an Nvidia GTX 1080 graphics card, running Windows 10.

The participant was seated in a Playseat Evolution Alcantara playseat (Playseats B.V., The Netherlands) aligned with the mid point of the 55′′ display screen (LG 55UF85). The screen resolution was 1,920 × 1,080 pixels, the frame rate was 60 and the refresh rate 60 Hz. The viewing distance was adjusted for each participant (so that they could place their hands on the steering wheel comfortably) and was approximately between 90 and 120 cm from the eye to the screen. The game was controlled with a Logitech G920 Driving Force steering wheel (Logitech, Fremont, CA). Steering wheel settings in Logitech Gaming Software 8.96.88 were: sensitivity 100%, centering spring strength 4 percent, and wheel operating range 900°.

Eye-tracking and physiological signals were collected and stored on an Asus UX303L laptop with Debian GNU/Linux 9 OS. Electrodermal activity (EDA) and blood volume pulse (BVP) were recorded at 128 Hz sampling rate using NeXus-10 (Mind Media B.V, Roermond-Herten, The Netherlands). For EDA, silver-silver chloride (Ag-AgCl) electrodes with 0.5% saline paste were attached to the medial side of the left foot with adhesive skin tape and gauze. The BVP (heart rate) sensor was attached to the left index toe of the participant. Eye tracking was measured with a Pupil Labs Binocular 120 Hz headset with a custom-built headband^4^.

#### 2.3.3. Flow Short Scale

To measure self-reported Flow, participants were asked to fill in the Flow Short Scale (FSS) after each trial (Rheinberg et al., 2003; Engeser and Rheinberg, 2008). FSS has 10 core items which load the subfactors *fluency of performance* (6 items) and *absorption by activity* (4 items); plus 3 items for *perceived importance*. The response format of FSS is a 7-point Likert scale ranging from *Not at all* to *Very much*. Higher scores on the scales indicate higher experienced Flow and perceived importance. Example items include “My thoughts/activities run fluidly and smoothly” (*fluency of performance*), “I do not notice time passing” (*absorption by activity*), and “I must not make any mistakes here” (*perceived importance*). See Supplementary Information for full English text and Finnish translation.

Cronbach's alpha for a 10-item scale including the *fluency of performance* and *absorption by activity* items was 0.92; Cronbach's alpha was 0.87 for the 13-item FSS scale including *perceived importance* (Rheinberg et al., 2003). FSS authors (Rheinberg et al., 2003) suggest using the 10-item scale (excluding *perceived importance* subfactor) as a measure of experienced Flow. For our data also, Cronbach's alpha was higher for the core 10- than for 13-item scale. Thus, the Flow scale used in our analyses was formed by averaging the items in the *fluency of performance* and *absorption by activity* subfactors. The *perceived importance* subfactor was used separately in some analyses (see section 3).

In addition to the 13 main items asked after every trial, participants were asked at the end of every session to report 3 more items measuring the fit of skills and demands of the task (Rheinberg et al., 2003). These items also had 7-point scales, e.g.,: “For me personally, the current demands are…(too low—just right—too high).”

There was no Finnish translation of the scale available, so it was translated into Finnish by the authors. Two of the authors (native speakers of Finnish, no formal qualifications for English-Finnish translation) first made translations independently; these translations were compared and revised, then reviewed by other Finnish-native authors, and revised.

### 2.4. Procedure

After recruiting, participants selected eight suitable dates within a 3-week period. All sessions took place between 8 a.m. and 7 p.m. at Traffic Research Unit, Department of Digital Humanities, University of Helsinki. In the first session, participants were informed about the procedure of the study and asked to fill in a background information questionnaire, including information on health, driving experience and gaming experience, and an informed consent form.

The sessions were managed by two research assistants at a time, who observed the measurement, out of participants' line of sight behind a partition wall, and took notes about possible confounding factors and problems within the session. In the beginning of each session participants filled in a session-wise questionnaire on the use of contact lenses, restedness, and medication, caffeine, and nicotine intake.

In sessions with physiological measurements (1 and 5 to 8), participants were dressed in physiological sensors and an eye-tracking headset, seated in the driving seat in quiet, low-light conditions for baseline measurement. They were asked to sit still for 5 min, looking at a dark blue screen, while baseline was recorded. After baseline recording, participants played five game trials, filling FSS after each trial. Eye-tracking and physiological signals were recorded during trials. In sessions 2–4, participants played five trials straight after filling in the session-wise questionnaire, without a baseline period. The FSS was filled after each trial. At the end of Session 8, the participants were debriefed and given the reward of culture vouchers.

### 2.5. Statistical Methods

All statistical data processing reported herein was implemented with R platform for statistical computing (R Core Team, 2014). Where possible, exact corrected *p*-values are reported; inequalities are reported where exact values were not available. All *p*-values were corrected for multiple comparisons using Bonferroni-Holm. For all simple correlations we calculated Pearson's correlation coefficient, because all data in these tests were shown to be normally distributed by Shapiro-wilk tests and associated Q-Q plots.

For RQ1, participant-wise linear regression models were fitted using *lm* function in R, which also supplies *R*^2^ values. The same approach was used to fit the “grand model” to group-wise data (i.e., pooled participants).

For RQ2, we obtained the independent variable as follows. For each participant and for each trial (40 trials in total), we subtracted predicted trial duration (*y*-value of power-law performance line) from observed trial duration, thus obtaining power-law model residuals, in units of log(sec). We refer to these within-participant trial-duration residuals as *deviation scores*, because they represent how much each observed trial duration deviates from the duration predicted by the model. Note, residuals are in the space of log-transformed trial durations in seconds and are therefore equivalent to ratio of performance in seconds. So for similar deviation scores from two trials, the later deviation represents a larger (or equal) effect in seconds.

Specifically, we first fit a linear mixed model with non-standardized Flow scores as the dependent variable, deviation scores as the predictor, and participant (numerical participant identifier ranging from 1 to 9) as a random factor with both random intercept and slope. This approach was chosen to handle the non-independence of data points within participants (see Bates et al., 2015).

Note that that there is no consensus on the best way to obtain *p*-values or estimates of effect sizes from linear mixed models. We have treated the *t* statistic as a *z* statistic using a standard normal distribution as a reference, and followed the method by Nakagawa and Schielzeth (2013) to obtain pseudo-*R*^2^ values. Another way to statistically evaluate the significance of these results is via the binomial distribution: The (two-tailed) probability of 9 negative slopes (should the probability of a negative slope per participant be 0.5, that is, fully random) is *p* = 0.007.

## 3. Results

All participants completed the task (40 trials in total). Average trial duration was 186s (SD 18.2 s, min 162.2 s, max 300.1s). Average number of collisions was 17.8 (SD 4.9, min 5, max 40). Average trial velocity ranged between 1.37 and 2.54 units per step (mean 2.23, sd 0.19). Maximum instantaneous speed was 3.6 and minimum 1.06. The Supplementary Information provides comprehensive data on performance-related features, such as trial duration, along with correlations between them; also it includes further details on participant self-report and background, plus validation of our main result for RQ2.

### 3.1. RQ1: How Does Performance Change Over Time?

**Figure 3:**
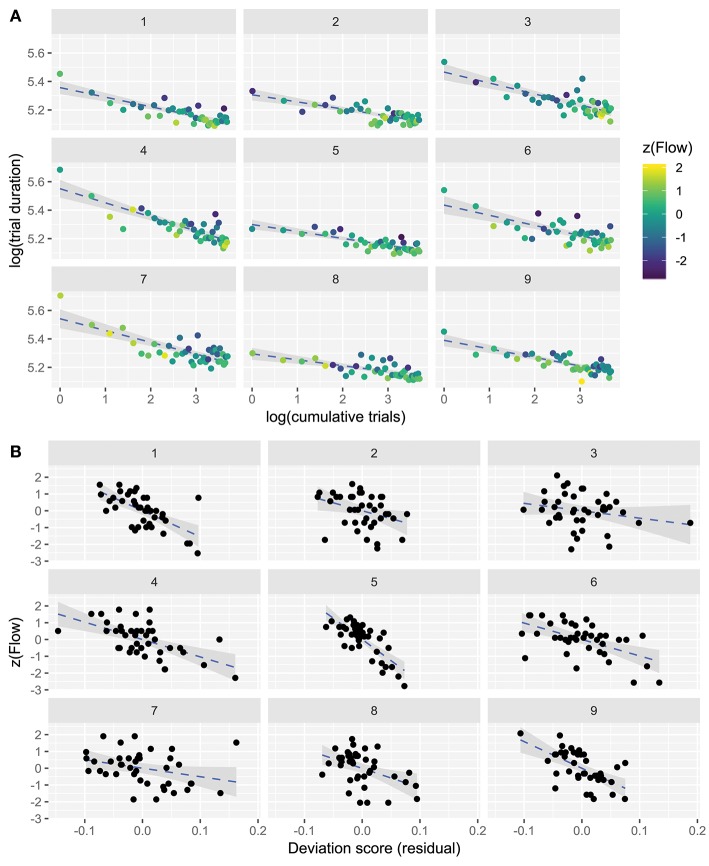
**(A)** Participant-wise data showing logarithm-transformed performance and Flow self-reports in the speeded steering task. Ordinate shows log-duration of trials, abscissa shows log-cumulative trial count. Dashed blue lines fitted to the data are power-law learning curves, which transform to linear in log-log space. 95% confidence intervals around the slope are grayed. Flow scores (z-scored) are indicated by color. **(B)** Participant-wise deviation scores (observed trial duration minus predicted trial duration) plotted against Flow scores for each participant, and fitted by linear models. 95% confidence intervals around the slope are grayed.

What is the form of the learning curve, does it consistently improve e.g., as a power law of practice (Newell and Rosenbloom, 1982)? A power-law curve transformed to log-space will be linear. Thus, to investigate whether participant behavior follows a power law, we fitted a linear model in log-log space (log-transformed dependent and independent variable) of trial durations as a function of cumulative number of trials, for each participant separately. Figure 3A shows this log-log performance data for each participant in each trial. Blue dashed lines indicate the power-law LC. Distance of points from the line (residuals) indicate the deviation of each trial from predicted learning: points above the line indicate longer duration (worse performance) than predicted by the LC, and vice versa.

All participant-specific log-log models had negative slopes, which indicates that with experience each participant learned to play better (obtained faster trial times). The variation in intercepts reflects disparity in participants' initial skill levels, and the variation in slopes the different learning rates. The individual intercepts and slopes of the models are presented in Table 2. A grand model was also fitted for all participants, and cumulative number of trials explained 39.6% of variance in trial durations. As the performance generally improves with cumulative trials, in agreement with a power-law of learning model, the explained variance can be ascribed to learning.

**Table 2:** Individual learning rate parameters (cols 2–4), Flow (cols 5–6) and perceived importance (P.I., cols 7–8) scores. Last row shows group-mean values of each column.

**Participant**	**Intercept sec**	**Intercept log**	**Slope**	**Flow mean**	**Flow SD**	**P.I. mean**	**P.I. SD**
1	213	5.36	–0.067	5.10	0.51	3.53	0.57
2	202	5.31	–0.049	5.18	0.39	4.29	0.47
3	237	5.47	–0.077	5.36	0.64	2.03	0.50
4	257	5.55	–0.099	4.40	0.39	4.12	0.46
5	200	5.30	–0.049	5.44	0.88	5.16	0.64
6	230	5.44	–0.071	5.22	0.82	4.33	0.56
7	255	5.54	–0.083	4.69	0.53	2.22	0.53
8	198	5.29	–0.041	4.94	0.90	3.67	0.70
9	219	5.39	–0.059	5.25	0.79	4.62	0.55
Group mean	223	5.41	–0.066	5.06	0.65	3.77	0.55

To confirm that a power law model gives a good approximation of learning, we compared its model-fit criterion against the fit of an exponential curve model (see Supplementary Information for details). While both models had good fit, the power law model was slightly better.

RQ1 can thus be answered: *the task was learned and the LC fit well to a power law model*. Given these positive answers, we may assume that the model provides a useful statistical estimate of performance expectation, i.e., how well the participants expect to perform can be estimated from the model.

### 3.2. RQ2: How Is Flow Related to Performance?

**Figure 4:**
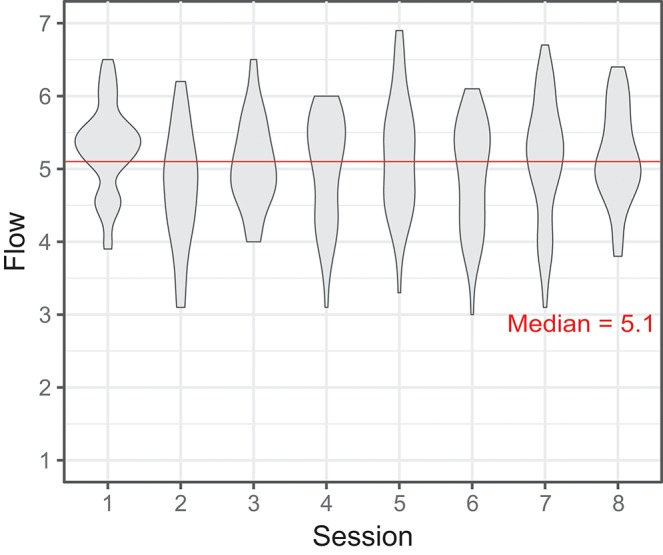
Violin plot representing participants' self-reported Flow in sessions 1–8 (per-session Flow = mean of five trials. The self-report items are given in Supplementary Information, the scale was 1–7).

Participant-wise mean Flow and LC slope were related but not significantly correlated (Pearson's correlation coefficient *r* = 0.6, *p* = 0.6, *N* = 9). Since we have established that performance improves over sessions, we also used session number as a simple proxy of performance improvement. Figure 4 shows the group-wise distribution of Flow scores plotted against sessions: clearly, there is no effect of session on group-wise median Flow (Pearson's *r* = -0.12, *p* = 1.0, *N* = 8). We found no significant effect of session type (sessions 2–4 vs. sessions 1, 5–8) on Flow scores [*F*_(1, 8)_ = 3.18, *p* = 0.11], by repeated measures ANOVA.

**Figure 5:**
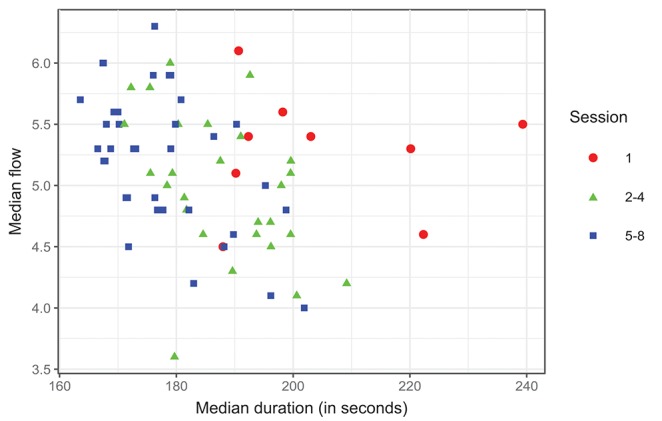
Duration and flow over sessions grouped: 1 (Introduction+physiological measurements), 2–4 (training), 5–8 (physiological measurements), *N* = 72.

Next, we calculated the group-wise correlation of median duration and median Flow, separately for each session. The relationship between duration and Flow was intermittently significant *before correction for multiple comparisons*, but not after, and with no particular trend (range of Pearson's *r* = [-0.05 … -0.74], *p* = [0.2 … 1.0], *N* = 9 for all). These results suggest that higher Flow was sometimes associated with lower trial durations (i.e., better performance), but not strongly and not systematically. If we group sessions by condition (introduction = 1, practice = 2–4, main test = 5–8), we can visualize the evolution of performance against Flow more clearly than by plotting each session individually, see Figure 5.

The relationships between global (over all responses) Flow and performance appear weak, but we also wish to examine local Flow for each trial separately. The points in each subplot of Figure 3A are colored according to Flow self-reports made after each trial, in a standardized range (original scores transformed to *z*-scores). The highest Flow scores are yellow, the lowest are navy-blue. Interestingly, this figure reveals at a glance that the points lie above and below the log-transformed power-law line in good agreement with the level of experienced Flow: worse performing trials (data-point above the line) tend to be more blue (Flow scores below the participant-wise mean), and better performing trials tend to be more yellow (scores above the mean). In other words, it seems that whenever participants were performing *better than predicted by the power-law line*, they were experiencing more Flow, and *vice versa*.

We evaluated whether this effect was robust and statistically significant. For each participant, we correlated their *deviation scores* (signed residuals from the power-law model) with their Flow scores, using a linear mixed model (see section 2). This model was statistically significant (deviation score β = -8, *t* = -4.36, *p* = 0.002) and the relationship is shown in Figure 3B (Flow scores are standardized). The conditional pseudo-*R*^2^ value for this model was 0.47, corresponding to a correlation of 0.68, so that the model explains ~47% of Flow score variability.

Thus, *high Flow scores are associated with better than predicted results* (trial durations below the predicted performance line), and *vice versa*. The strength of this association per participant follows from the strength of the correlation, and overall the model has large effect size.

As can be seen in Figure 3B, the trend was clearly negative for 7 out of 9 participants, while for two participants, 3 and 7, the trend was similar but the relationship was weaker. Notably, these two participants also reported lower scores on perceived importance: mean scores for these participants were 2.03 and 2.22, whereas the overall mean was 3.77 (see Table 2). However, the group-wise interaction between perceived importance scores and deviation scores was not statistically significant.

RQ2 can thus be answered: Flow was not consistently and robustly related to improvement in task performance with the skill acquisition occurring over 2 h of practice. It was, however, consistently related to whether performance was *better (or worse) than predicted* given the participant LC. Moreover, this effect might be moderated by self-reported perceived importance of the steering task (more data would be required to clarify).

## 4. Discussion

We present a longitudinal experiment of Flow in a game-like high-speed steering task where task performance is easily parameterized and its relation to Flow analyzed. To induce Flow, the game was designed to hold the balance between skill and challenge constant: the difficulty of the game continually adapted to the skill level of the participant.

The results show the game was clearly Flow-inducing: mean Flow across sessions was reported as 5.1 (out of 7) on the FSS. This relatively high and stable mean Flow “baseline” induced by the game could be construed as reflecting the meeting of skill and challenge C1–3 by design.

We further found that Flow was not associated with gaining experience and skill in the game—our participants did not reliably report more Flow even as they learned, session by session, to complete the trials faster. This supports the theoretical position that Flow is elicited by the balance of skill and challenge, but show that Flow is less sensitive to the absolute level (within a task) of skill or challenge. This fits with the models which are more lenient regarding skill/challenge level: the original Flow Channel model and the latest Flow intensity model. The Quadrant/Octant models, which require “above-average” skills and challenges for Flow, are only supported under the assumption that this above-average reference level is task-specific and dynamically adjusted in step with the learning curve. Otherwise, if the reference level is fixed, then based on the Octant model experience of Flow would be predicted to increase along with the increase of skill level (and demand) during our longitudinal measurements of participant learning. In other words, when skills and challenges increased from a fixed reference, participants should have felt further “north-east” of the model midpoint where Flow bottoms out, and thus be more likely to report Flow and assign it greater intensity on a reporting scale. This was not observed. Therefore, our results do not fit the predictions of the Quadrant/Octant models *under the assumption of a fixed-demand reference level*. See also Keller and Landhäußer (2012) for critical discussion of these models. In absence of an independent motivation for such adjustment hypothesis it must be considered somewhat *ad hoc*. So, we suggest that—in order to incorporate the present findings—the octant model should be developed to provide a valid set of assumptions to support clear conclusions about the reference level.

### 4.1. Mechanisms of Learning and Flow

We also showed that higher trial-wise Flow (trials with higher self-reported Flow) was associated with trial durations shorter than expected by the power law LC model, and vice-versa. Thus, Flow for each trial was higher-than-average or lower-than-average in agreement with task performance that was better or worse than expected (at the current level of skill). This stands in contrast to how mean Flow remained stably elevated across sessions. In other words, learning to play the game did not itself increase Flow; rather, the game induced a fairly high level of mean Flow, and trial-wise variability of Flow was correlated with better or worse than statistically-expected performance.

#### 4.1.1. Alternative Explanations of Trial-Wise Results

It is a novel observation that trial-wise Flow relates to fluctuations of performance around the level expected from the learning curve. It is interesting because higher-than-expected performance in an individual trial can plausibly indicate either higher skill (e.g., better concentration), or lower challenge (easier-to-negotiate random placement of obstacles). Both are deviations from the average skill-challenge balance, yet may be associated with higher Flow. Either way, *our result undermines the assumption of Keller and Landhäußer (**2012**)'s Flow Intensity model that the direction of skill-challenge ratio can be ignored: our results show that Flow is elevated when skills exceed challenges*.

There are naturally several alternative explanations for this result. One possibility is that performance on some trials is enhanced by increased Flow during the trial—i.e., participants perform better when they “get into Flow.” Another possibility is that the randomly-generated geometric layout of each trial might be more or less easy to negotiate; the “easier” trials would afford faster performance. Such random fluctuations of task difficulty could shift the skill-challenge ratio closer to one that the participant finds Flow-inducing. Alternatively, participants may be more likely to report higher Flow (after the fact) on a more successful (hence, more rewarding) trial, because they see their score before they complete the FSS. From these three alternatives, we find the first one the most convincing, because (A) comparing first to second, we believe that whatever biological and psychological mechanisms might be underpinning Flow will tend to create greater variation than the randomly-generated track layout; and (B) comparing first to third, the game design undermines the third alternative (see Limitations below). Ultimately however, the present paradigm cannot conclusively support any one alternative.

#### 4.1.2. Cognitive Mechanisms of Flow

How can our approach and the present results be helpful to understand the mechanisms generating the Flow experience?

Methodologically, this study follows a *time series* approach rarely used in Flow research, by looking at changes in Flow over time, with relatively highly frequent and non-independent repeated measurements. This contrasts with much prior work which treats Flow as a relatively stable property, and allows us to look at on-task learning.

Learning implies skill increase, which (by game design) implies challenge increase, which (by design of Quadrant/Octant models) together imply Flow increase. As discussed, for such “state Flow” models the assumption of the fixed reference frame leads to the prediction of increased Flow with skill acquisition, which is not supported by our data. It is thus not straightforward to reason about the evolution of Flow, or learning and Flow, based on these models, before resolving the crucial issue of the reference level for above-average task demand and skill.

For the Flow intensity model this particular problem does not arise (Keller and Landhäußer, 2012). However, the relation of learning and Flow is not entirely straightforward here, either. Increased skills should eventually increase task demands (because skill-learning increases access to the task's deeper levels of challenge), and thus perceived fit of skills and task demands. We did not assess perceived value directly, but it is plausible that time investment in and enjoyment of the game (as indicated by high mean Flow) would *also* increase the subjective value of the activity. If this is the case, again higher Flow should be elicited in step with increasing performance. This was not observed.

Overall, the lack of mechanistic hypotheses about the processes underlying the proposed dependencies in the Flow models make it difficult to make definite predictions in novel tasks, especially ones with changing task demands and skill, such as here. Prior work has provided some (neuro)cognitive, information-processing views on Flow (Marr, 2001; Cowley et al., 2008; Šimleša et al., 2018). Such work could provide an approach to make cognitive hypotheses about Flow, but these models have not been empirically tested, so it is unclear which (if any) to follow.

The aim of future work should then be to find out: what cognitive processes are *specific* to Flow-inducing task performance (in different stages of learning), but also *general* to multiple performance-domains. By so doing, we can in future attempt to clarify empirical observations by reference to a distinct cognitive theory of how Flow is generated.

#### 4.1.3. Flow and Task Complexity

A possibly useful novel way to view Flow and learning is via task complexity. Csikszentmihalyi (1999) proposed that Flow should be possible in *any* task, complex (e.g., car driving) or simple (e.g., dish washing). But Keller and Landhäußer (2012) also proposed that Flow depends on perceived task value as well as challenge–skill balance. One way to resolve these ideas is to consider that the individual can *introduce* complexity (or value) to their activity if they appear to have exhausted that task's potential to challenge them. Nakamura and Csikszentmihalyi (2002) suggest such an exploratory mechanism to explain how individuals maintain Flow in complex tasks: “As people master challenges in an activity…to continue experiencing Flow, they must identify and engage progressively more complex challenges.” The corollary for simple tasks is that individuals *create* complexity, e.g., with self-defined goals (Rauterberg, 1995). For example, a similar state to Flow, called the Zone, has been reported for machine-gambling addicts whose pastime is in fact skill-free, but who nevertheless *believe* that they are skilled (Schull, 2014).

In summary, complex tasks have deep structure to be learned, requiring non-trivial skill acquisition for any duration of learning and thus a shallow LC (learning is slow). Importantly, the skill level does not quickly peak, such as with simpler tasks like washing the dishes, where Flow might be obtained but cannot strongly interact with learning (without self-created complexity). Learning comes into play when we consider that the same task can appear at first simple and later complex, e.g., as our experiment game.

### 4.2. Limitations and Future Work

Our study had a small convenience sample because (a) the recording paradigm was extensive (around 8 h of contact time), and (b) it was to some degree an exploratory study; both implying the need to constrain datasets to tractable sizes. It is worth noting that while we recruited only 9 participants, we collected a significant amount of behavioral and physiological data for each participant over a course of 2 weeks and 8 recording sessions. This amounts to a quite rich dataset allowing us to dig deep into the underpinnings of skilled high speed steering and Flow. Moreover, given the fact our experimental paradigm has not been used previously, we could not *a priori* easily estimate the statistical power required to discover significant effects. However, our results ended up being both strikingly *similar* and *strong* within each participant, suggesting that collecting a larger sample size would likely have *not* changed the pattern of the results or provided more in-depth insights. Regardless of the justifications, the sample size and recruitment method are minor limitations to be remedied in future work. For example, gender is known to affect performance visuomotor tasks (Feng et al., 2007). However, although there is a gender imbalance in our sample, the factor gender is confounded by background variables including gaming and driving experience, masking the true effect of gender. In order to properly study gender differences we would need to tailor our recruitment to that purpose. On the other hand, the result of Feng et al. (2007) suggests that gender differences might anyway balance out over the course of learning such a task as ours. Finally, because it lacks experimental manipulations the study cannot make strong causal claims, which could be improved by recording separate conditions of the game task, e.g., with varied difficulty levels.

As stated above, there are different plausible explanations of the main result linking Flow and performance. If FSS reports were indeed influenced by seeing the score beforehand, this should be considered a design limitation. However, the game gives such clear and direct feedback on performance (i.e., after collisions), that it is likely that self-assessment of performance would be similar with or without seeing the score. The score can thus be considered just a reinforcement of the perception of fit between skills and demands, which is anyway a required part of Flow reporting (Keller and Landhäußer, 2012). There is a possibility that Flow states could be affected by wearing eye-tracking and physiological equipment in some of the sessions. However, there was no difference in the level of subjective Flow reports in these sessions compared to other sessions.

Trial-by-trial analysis is limited by the Flow self-report which only has one data point per trial. It would be more powerful to analyse *inside* each trial. In our data, self-reported Flow is a point model of an entire trial, for which the participant knows their score. Self-reported Flow is thus an after-the-fact report, and could be criticized for not capturing the in-the-moment *experience of Flow*, which might fluctuate greatly during a trial. Analysing the fluctuation of Flow-experience requires us to model individual actions and/or their outcomes, and sample Flow during performance. This is a difficult challenge, because paying attention to one's phenomenal state might easily disrupt the very processes sustaining that state, especially for Flow which is unreflective by definition. Future work should aim to model the conditions of Flow (C1–3) in real-time, while simultaneously recording participant physiology, to uncover in greater detail the relationships involved. The existing dataset will be used for this purpose, in a pending report on the biosignals recorded with high temporal resolution, primarily electrodermal activity.

Future work should also look into individual differences in learning (or cognitive) styles. For example, ample evidence suggests people can be roughly placed on a continuum of verbal as opposed to visual learners. These learning styles, in turn, have been positively linked to cognitive abilities on either verbal or visual tasks (e.g., Choi and Sardar, 2011; Knoll et al., 2017). Since the task used in our study was highly visual (there were no verbal cues nor audio), learning in it might be moderated by players cognitive learning styles. For example, perhaps those with a propensity for visual learning find it easier to get into flow and thus perform better. Therefore, an interesting avenue for further research using our driving task is first dividing people into verbalizers and visualizers, and then seeing if visualizers in particular find the game flow-inducing.

In terms of possible applications, game-induced Flow has been studied in the context of technology-enhanced learning (TEL) games (e.g., Cowley et al., 2014), but the style of activity in such games tends to be rather more complex than the driving task reported here. Use of such simple tasks for TEL games has been reported (Cowley et al., 2011), but it remains unclear what higher-learning benefit is derived from the Flow induced by the TEL game (Cowley and Bateman, 2017). In summary, there remains a large conceptual gap between what is known about task-learning and Flow, and how to make use of Flow-inducing tasks for higher learning applications.

### 4.3. Conclusion

We report results that self-reported Flow in a novel, challenging, and engaging high-speed steering task relates to trial-by-trial task performance relative to the learning curve: “better than expected” trials have higher Flow scores, and “worse than expected” trials have lower scores. The average level of self-reported Flow was high, as the game was specifically designed to meet the main preconditions of Flow, including balance of current skill and challenge. Perhaps surprisingly, Flow did not seem to change with global skill development or improvement in task performance.

These results show that: (1) If a reference level is important (as the octant model requires), it is so on a trial-wise scale. In other words, the reference level is task-specific and is continually adjusted during skill acquisition, following in step with the individual's own learning curve; (2) Contrary to the intensity model (Keller and Landhäußer, 2012) the direction of challenge-skill deviation cannot be ignored. Our study highlights a need for models of Flow to be developed in a way that better captures Flow dynamics, over the range of skill acquisition from novice to expert, than the state-like models of the phenomenal psychology tradition (Moneta, 2012).

Understanding how phenomenological experiences, such as Flow, relate to task performance is an important topic for understanding human motivation and performance. This study contributes to this goal and we hope will inspire more inquiry into the dynamics of the Flow experience in different stages of learning a skill.

### 4.4. Context

The three senior authors conceived and guided the work. BC (the lead author) has studied computer gameplay from the perspective of performance, psychophysiology and emotional experiences. OL (the last author) has studied visuomotor skill in the domain of steering, and with JV he designed the “Flow-inducing” high-speed steering game. JP (the second author) has studied emotions and decision-making in games of skill and chance, and written on the similarities and differences between the Zone and Flow phenomena. Because these authors share a keen interest in understanding the development of expertise and the phenomenon of Flow, it was decided to join forces and put together a team of researchers to develop the present experiment on the basis of the steering game designed earlier. The other authors (all more junior graduate students) were recruited to work as part of their studies. This experiment is part of a larger effort to initiate a line of research into the neurocognitive processes underlying Flow and expert performance, combining experimental, psychophysiological, and computational methods.

## Data Availability

The R code and data used to produce all analyses and figures is permanently available online at https://doi.org/10.6084/m9.figshare.7268387.

## Ethics Statement

Participants were briefed and provided written informed consent before entering the study, and were aware of their legal rights. The study followed guidelines of the Declaration of Helsinki and was approved by the University of Helsinki Ethical review board in humanities and social and behavioral sciences (statement 31/2017; study title MulSimCoLab).

## Author Contributions

OL, JP, and BC conceived the study. OL and JV designed the gameplay. JV developed the game software. BC and TT designed and implemented the data collection. NL, TT, PP, RF, V-PI, and JP translated the FSS. All authors participated in decisions on the experiment specifications. RF, V-PI, NL, PP, and TT conducted the experiment. BC, V-PI, TT, RF, PP, NL, JP, and OL analyzed and interpreted the results. BC, JP, and OL drafted the paper. All authors participated in writing and reviewing, and approved the manuscript.

### Conflict of Interest Statement

The authors declare that the research was conducted in the absence of any commercial or financial relationships that could be construed as a potential conflict of interest.

## Footnotes

^1^As an example of a universal benchmark, the maximum propagation speed of neural signals along an axon sets a limit on response times.

^2^For game video see https://doi.org/10.6084/m9.figshare.7269395.v1

^3^The game code as used herein is permanently available under open source license at https://doi.org/10.6084/m9.figshare.7269467

^4^For technical details, see https://zenodo.org/record/1246953#.XJT-Ki10eqA
