# Supplementary material for: Flow Experiences During Visuomotor Skill Acquisition Reflect Deviation From a Power-Law Learning Curve, but Not Overall Level of Skill
Source: Front Psychol. 2019 May 15;10:1126. doi: 10.3389/fpsyg.2019.01126 (PMC6530424; doi:10.3389/fpsyg.2019.01126)
Supplement: Supplementary file 1 [file Data_Sheet_1.PDF]

Flow Short Scale, in English with Finnish Translation

Core items of Flow: *fluency of performance* (2, 4, 5, 7, 8, 9) and *absorption by activity* (1, 3, 6, 10):

1.	I feel just the right amount of challenge	Peli tuntui juuri sopivan haastavalta
2.	My thoughts/activities run fluidly and smoothly	Pelasin sujuvasti
3.	I do not notice time passing	En huomannut ajankulkua
4.	I have no difficulty concentrating	Pystyin hyvin keskittymään
5.	My mind is completely clear	Mieleni oli selkeä
6.	I am totally absorbed in what I am doing	Uppouduin täysin pelaamiseen
7.	The right thoughts/movements occur of their own accord	Löysin oikeat liikkeet kuin itsestään
8.	I know what I have to do each step of the way	Olin koko ajan tilanteen tasalla
9.	I feel that I have everything under control	Tunsin hallitsevani tilannetta
10.	I am completely lost in thought	Syvennyin peliin täysin

Extra items for *perceived importance*:

11.	Something important to me is at stake here	Koin pelissä onnistumisen tärkeäksi
12.	I must not make any mistakes here	Minusta tuntui siltä, etten saisi tehdä yhtäkään virhettä
13.	I am worried about failing	Pelkäsin epäonnistuvani

Extra items for the fit of skills and demands:

14.	Compared to all other activities which I partake in,this one is... (easy/difficult)	Verrattuna muihin tekemiini asioihin, tämä on... (helppoa/vaikeaa)
15.	I think that my competence in this area is... (low/high)	Osaamiseni taso on... (matala/korkea)
16.	For me personally, the current demands are... (too low/just right/too high)	Pelin vaativuus on tällä hetkellä minulle... (liian matala/sopiva/liian korkea)

Descriptive Statistics and Visuals

Table 1: Descriptive statistics of the game performance measures.

Variable	Description	Mean	Median	SD	Min	Max
min_velocity	Minimum velocity of a trial	1.58	1.6	0.06	1.06	1.6
max_velocity	Maximum velocity of a trial	2.82	2.81	0.36	1.62	3.6
end_velocity	End velocity of a trial	2.72	2.78	0.4	1.14	3.59
avg_velocity	Mean velocity of a trial	2.23	2.26	0.19	1.37	2.54
Collisions	Number of collisions in a trial	17.81	17	6.59	5	40
speed_drops	Number of speed drops in a trial	12.57	12	3.96	4	28
duration	Duration of a trial (s)	186.07	181.99	18.24	162.15	300.1

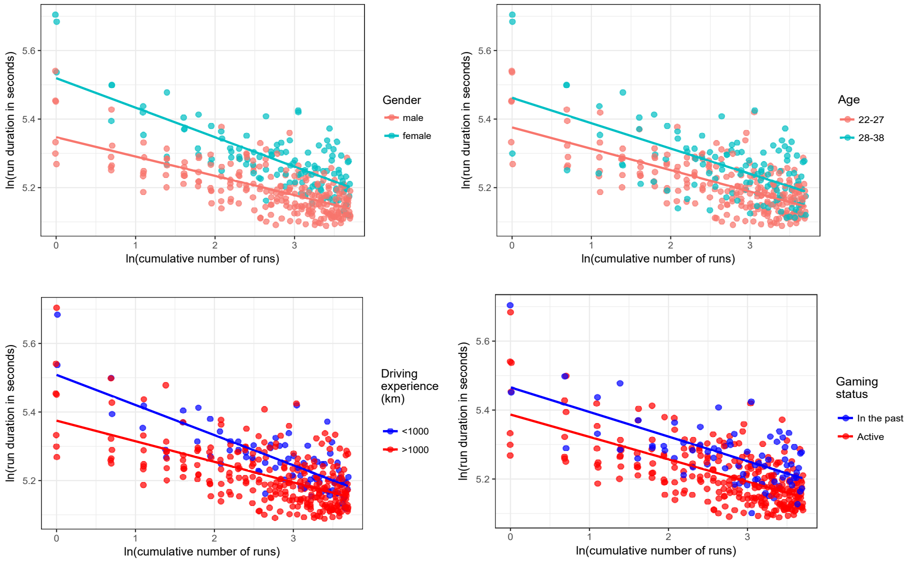

Figure 1: Visualisation of performance data coloured by demographic grouping factors: *top-left panel*, gender; *top-right panel*, age; *lower-left panel*, driving experience; *lower-right panel*, gaming experience. Each plot displays log-transformed cumulative trials and trial duration, thus representing a log-log space, where linear regression lines fitted to data subgroups are power law models in linear space.

Table 2: Descriptive statistics for fluency of performance and absorption by activity for each participant.

	Participant	Mean	Median	SD	Min	Max
Fluency						
	1	4.93	5.00	.62	3.17	6.00
	2	4.90	4.83	.47	3.83	5.67
	3	4.90	5.00	.84	3.00	6.67
	4	4.10	4.00	.56	3.00	5.33
	5	5.15	5.50	1.01	2.67	6.50
	6	5.11	5.25	.92	2.83	6.50
	7	4.35	4.33	.58	3.17	5.50
	8	4.74	5.00	.92	2.50	6.33
	9	5.24	5.00	.86	3.33	7.00
Absorption						
	1	5.37	5.50	.45	4.25	6.25
	2	5.60	5.75	.37	5.00	6.00
	3	6.04	6.00	.43	5.25	6.75
	4	4.85	4.88	.39	4.00	5.75
	5	5.89	6.0	.80	3.50	7.00
	6	5.37	5.50	.74	3.50	6.75
	7	5.19	5.25	.54	4.25	6.25
	8	5.24	5.50	.95	3.00	6.75
	9	5.27	5.13	.72	4.00	6.75

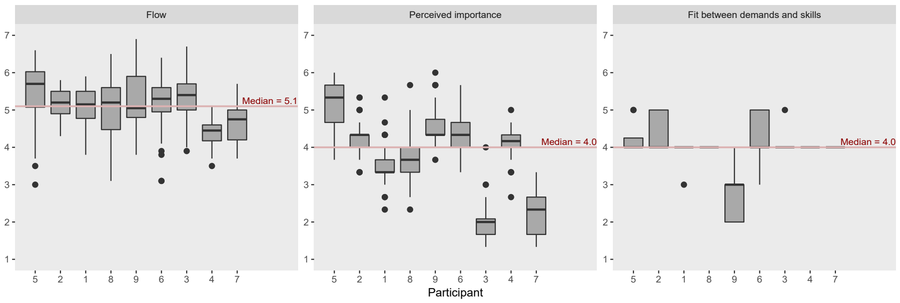

Figure 2: Participant-wise boxplots for Flow Short Scale measures, *left panel*: Flow (absorption & fluency), median 5.1, min 3, max 7; *center panel*: perceived importance, median 4, min 1, max 6; *right panel*: perceived fit of demands and skills, median 4, min 2, max 5. In each plot, boxes are ordered left-to-right by the game performance (mean trial duration) of participants, i.e. from best performer #5 to worst #7.

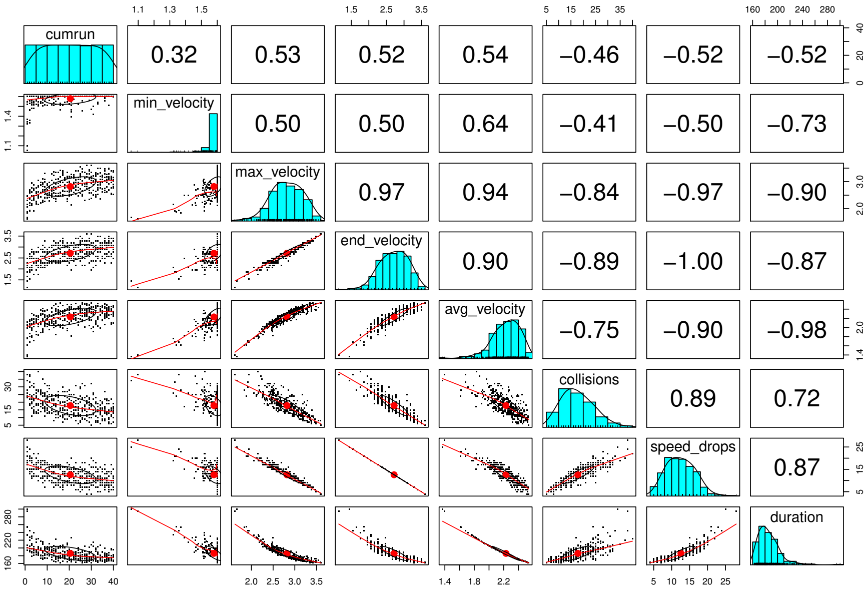

Figure 3: A scatterplot matrix of game performance measures. The diagonal of the matrix displays distributions (histograms) of each measure. The lower off-diagonal cells contain scatterplots, with loess-smoothed fit, of two measures from the corresponding row and column on the diagonal (panel above on the x-axis; panel to right on y-axis). The upper off-diagonal cells display corresponding Pearson correlation coefficients.

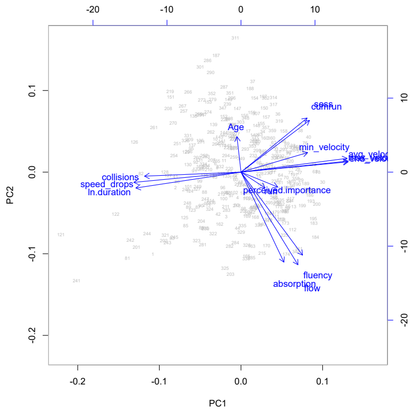

Variable	PC1	PC2
run (trial)	0.801	-0.088
min_velocity	0.221	0.109
max_velocity	0.356	0.062
end_velocity	0.356	0.057
avg_velocity	0.353	0.076
collisions	-0.321	-0.023
speed_drops	-0.356	-0.057
cumrun (trials)	0.228	0.289
age	-0.134	0.198
fss_fluency	0.204	-0.464
fss_absorption	0.143	-0.502
perc. importance	0.122	-0.083
Flow	0.189	-0.517
session	0.219	0.302
ln.duration	-0.349	-0.089

Figure 4: Summary of principal component analysis results: *Left panel* Biplot of principal components (PCs) 1 and 2; *Right panel* Component loadings for PC1 and PC2. Notice how Flow total score, and factors absorption and fluency, had distinctly negative loadings on PC2 compared to other variables. Also, Flow scores have almost completely orthogonal relationship to measures of game experience (session number and cumulative number of trials).

Additional Statistical Analyses

Permutation testing is another way to assess the statistical significance of the results presented in Figures 2A and B in the main text. For each participant, we first obtained their signed residuals from the power law model (see Figure 2A), which we then multiplied by their z-standardized Flow scores and summed across all trials.

This yielded d_o (observed deviation) scores, which have positive values when participants report higher Flow during better-than-predicted trials, *or* when they report lower Flow during worse-than-predicted trials. In other words, higher d_o scores reflect better agreement with the hypothesis that *participants report more Flow during better-than-predicted trials and vice versa for worse-than-predicted trials.* Thus, for each participant, we define the agreement of the Flow scores with the local performance prediction of the learning curve model using formula 1:

$$d = \sum_{x=1}^{40} \frac{f_x.(LCy_x - y_x)}{2} \quad (1)$$

where $x \in X$ is a trial from the set of all trials for a subject; and at each trial x: y_x is the observed performance, LCy_x is the performance value predicted by the learning curve model, and f_x is the standardised Flow score.

Next, we randomly permuted (over 10000 iterations) standardized Flow scores for each 40 trials for each participant and calculated a d_p (permuted deviation) score for each iteration. Finally, we obtained the distribution (histogram) of the permuted d_p values and computed the probability that the observed d_o values came from this distribution. For participants 1-9, these probabilities were <0.001, 0.012, 0.076, <0.001, <0.001, <0.001, 0.025, 0.002, and <0.001, respectively. See Fig. 5 for participant-wise permuted histograms of d_p values, with a vertical line marking the observed d_o value for each participant.

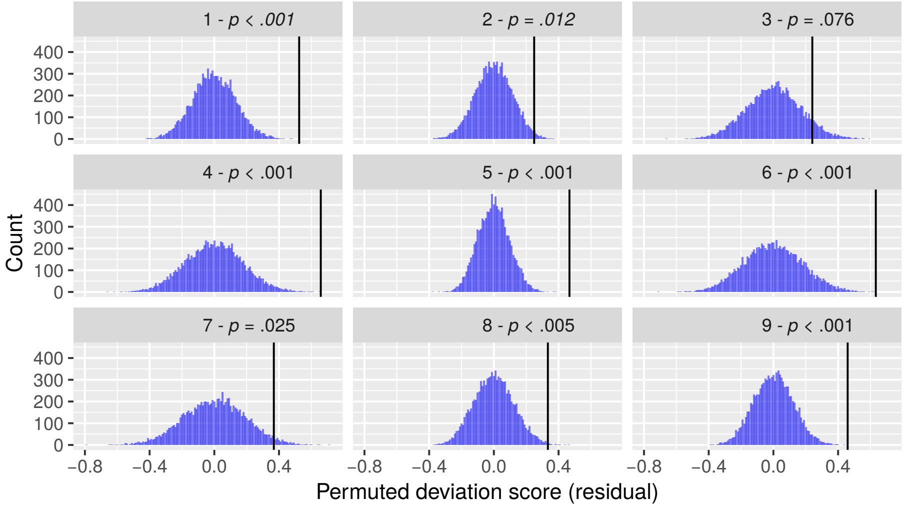

Figure 5: Participant-wise histograms of randomly-permuted deviation scores, with the actual observed deviation score marked as a black line. Here, we can see that the observed relationship between local Flow and performance is very unlikely to have been generated by a random process, even for those participants whose observed d_o value is closer to the random mean.

Finally, we compared the model fit criteria between the power law model (reported in the main text) and an exponential curve model as approximations of learning in the task. The exponential curve model had AIC and BIC values of -992.2 and -968.9, respectively, and a marginal pseudo-R^2 value of .289. The corresponding values for the power law model were -1118.6, -1095.3, and .397. Thus, while both models had a good fit, the power law model was slightly better across all fit indices. See Fig. 6 (panel A) for participant-wise data on the exponential curve model (transformed into linear space); panel B shows the untransformed performance data for comparison.

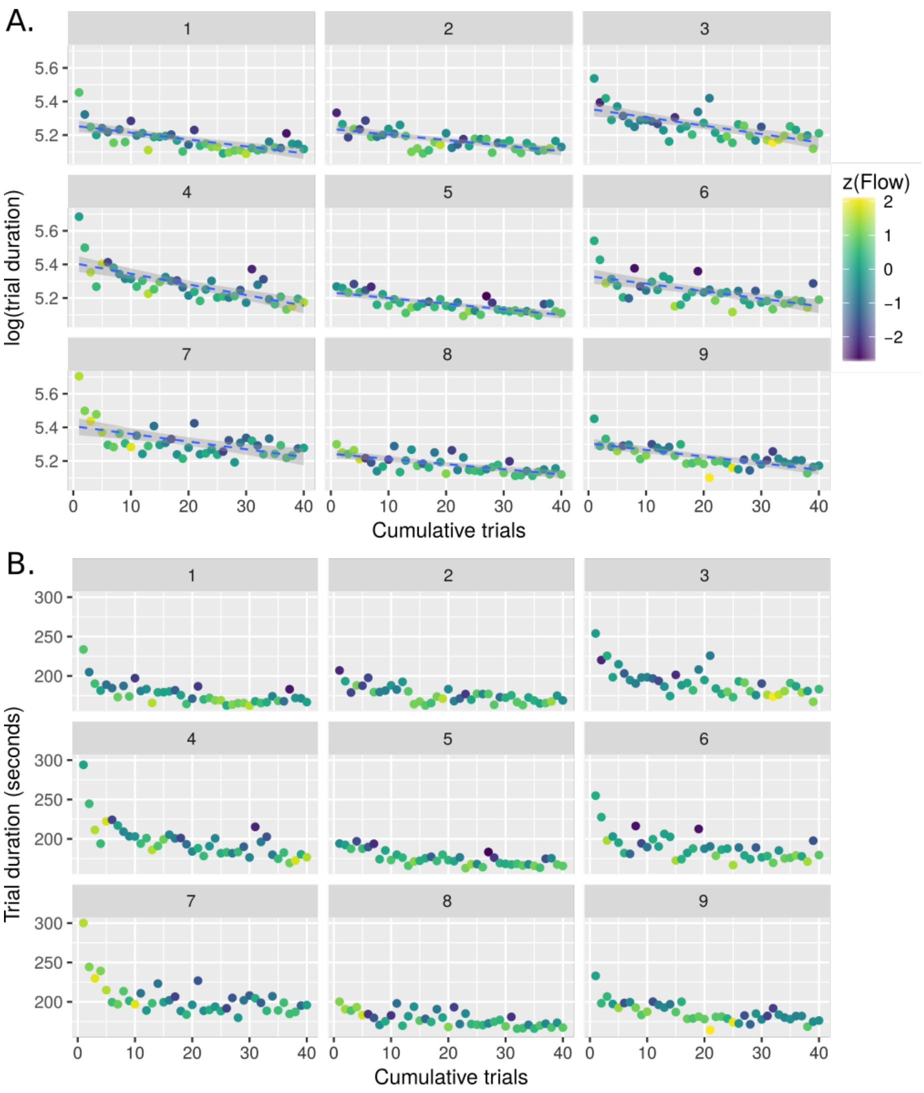

Figure 6: *Panel A*: Participant-wise data showing logarithm-transformed performance and Flow self-reports in the speeded steering task. Ordinate shows log-duration of trials, abscissa shows raw cumulative trial count. Dashed blue lines fitted to the data are exponential law learning curves, which transform to linear when the dependent variable is log-transformed

Panel B: Participant-wise data showing untransformed performance, otherwise similar to panel A.
